# Effects of a household air pollution intervention using liquefied petroleum gas stoves, continuous fuel distribution and behavioural messaging on dietary and sodium intake of adult women in Puno, Peru: a randomised controlled trial

**DOI:** 10.1017/S1368980023000320

**Published:** 2023-08

**Authors:** Carla Tarazona-Meza, Kendra N Williams, Gary Malpartida, Josiah L Kephart, Magdalena Fandiño-Del-Río, Suzanne Simkovich, Shakir Hossen, Marilu Chiang, Kirsten Koehler, William Checkley, Cardiopulmonary Outcomes and Household Air Pollution (CHAP) trial Investigators

**Affiliations:** 1 Department of International Health, Bloomberg School of Public Health, Johns Hopkins University, Baltimore, MD, USA; 2 Center for Global Non-Communicable Disease Research and Training, Johns Hopkins University, Baltimore, MD, USA; 3 Nutrition and Dietetics, Universidad Científica del Sur, Lima, Perú; 4 Division of Pulmonary and Critical Care Medicine, School of Medicine, Johns Hopkins University, 1830 E. Monument Street, Room 555, Baltimore, MD 21287, USA; 5 Biomedical Research Unit, A.B. PRISMA, Lima, Perú; 6 Department of Environmental Health and Engineering, Bloomberg School of Public Health, Johns Hopkins University, Baltimore, MD, USA

**Keywords:** 24-h Recall, Dietary intake, Liquefied petroleum gas, Clean cookstoves, Sodium, Household air pollution

## Abstract

**Objective:**

Household air pollution (HAP) is a widespread environmental exposure worldwide. While several cleaner fuel interventions have been implemented to reduce personal exposures to HAP, it is unclear if cooking with cleaner fuels also affects the choice of meals and dietary intake.

**Design:**

Individually randomised, open-label controlled trial of a HAP intervention. We aimed to determine the effect of a HAP intervention on dietary and Na intake. Intervention participants received a liquefied petroleum gas (LPG) stove, continuous fuel delivery and behavioural messaging during 1 year whereas control participants continued with usual cooking practices that involved the use of biomass-burning stoves. Dietary outcomes included energy, energy-adjusted macronutrients and Na intake at baseline, 6 months and 12 months post-randomisation using 24-h dietary recalls and 24-h urine. We used *t*-tests to estimate differences between arms in the post-randomisation period.

**Setting:**

Rural settings in Puno, Peru.

**Participants:**

One hundred women aged 25–64 years.

**Results:**

At baseline, control and intervention participants were similar in age (47·4 *v*. 49·5 years) and had similar daily energy (8894·3 kJ *v*. 8295·5 kJ), carbohydrate (370·8 g *v*. 373·3 g) and Na intake (4·9 g *v*. 4·8 g). One year after randomisation, we did not find differences in average energy intake (9292·4 kJ *v*. 8788·3 kJ; *P* = 0·22) or Na intake (4·5 g *v*. 4·6 g; *P* = 0·79) between control and intervention participants.

**Conclusions:**

Our HAP intervention consisting of an LPG stove, continuous fuel distribution and behavioural messaging did not affect dietary and Na intake in rural Peru.

Household air pollution (HAP) affects more than three billion people worldwide. HAP is generated by burning biomass fuels in open fire or traditional cookstoves, which occurs mainly in rural areas of resource-poor countries. Long-term exposure to HAP causes various negative health outcomes such as pneumonia, stroke and other cardiopulmonary diseases^(1–3)^. One strategy to reduce HAP is to replace biomass-burning stoves with stoves that run on cleaner fuels, such as liquefied petroleum gas (LPG), biogas, ethanol or electricity^(4)^. To our knowledge, few studies have explored the impact of different stove and fuel types on the nutritional content of meals, or how switching from a biomass stove to a cleaner fuel stove could change dietary patterns.

Understanding how dietary intake may vary by type of stove used is important given that diet may play a mediating role in the long-term effects of household fuel type on health outcomes^(5)^. One study showed that participants who received free biogas cookstoves had significantly higher dietary diversity than those who continued to use biomass cookstoves^(6)^; however, this study did not assess dietary intake. In a high-altitude area of Peru, not only did we find a higher dietary diversity in urban residents who more commonly used LPG fuel, compared to rural residents who primarily used biomass stoves, but also, that urban residents consumed more energy, protein and carbohydrates than their rural counterparts^(7)^. In this context, another study in Peru found that rural participants who used biomass fuels frequently preferred the flavour of food cooked with biomass stoves over that of food cooked with LPG^(8)^. This evidence suggests that people may prepare food and meals differently with different stoves. However, it is unclear from the existing literature whether changes in cooking practices are due to adoption of a new stove or differences in socio-economic status that typically correlate with cleaner cooking technologies^(9)^. Additionally, it remains unknown whether people will modify their dietary intake to improve a perceived diminished flavour of food cooked with cleaner fuel stoves, such as by adding extra salt.

Evidence about the potential effects of clean fuel interventions, like LPG, on dietary and Na intake are needed to help decision-making at the policy level. Our study aimed to fill this knowledge gap by comparing the dietary and Na intake of adult women who received free LPG stoves and fuel and control participants who continued to cook with biomass stoves within a randomised cleaner fuel trial. We also compared macronutrient and Na intake to nutritional recommendations in our study setting.

## Methods

### Study setting

Our study was conducted in rural communities of Puno, which is located in the southwestern Andean region of Peru bordering Bolivia at 3825 meters above sea level. Given its high altitude, average temperatures in Puno are cold, ranging between 5·9 and 9·8°C^(10)^. Most rural residents are farmers who grow crops such as potatoes, quinoa, fava beans and other tubers. The rainy season is from December to March, and the peak of the harvest season is in April and May. Most people speak Aymara or Quechua in addition to Spanish. Cooking is typically done with dung and wood in open fire or traditional biomass stoves, although access to LPG is increasing given recent governmental efforts^(11)^.

### Study design

This is an ancillary study of the Cardiopulmonary outcomes and Household Air Pollution (CHAP) trial. Details of the CHAP trial have been published elsewhere^(12,13)^. Briefly, we enrolled 180 adult women between the ages of 25 and 64 years who were the primary cook in their household and reported cooking daily with biomass fuels. We enrolled women from rural communities in Puno, Peru, and randomised every 14–16 participants each month in a staggered manner between February 2017 and February 2018. Participants were assigned to intervention or control in a 1:1 ratio. Eligibility criteria were described elsewhere^(12,13)^.

Intervention participants received a three-burner LPG stove (Industrias SURGES, Juliaca, Peru), LPG refills delivered as needed (approximately every 2 weeks) for 12 months and behavioural reinforcement for exclusive LPG use. Control participants continued cooking with biomass. Before receiving their LPG stove, participants in the intervention group attended a demonstration in which field workers prepared a local dish with the LPG stove to teach participants about safe operation and maintenance of the LPG stove and logistical benefits of LPG on health. We monitored the use of both LPG and biomass stoves in control and intervention households during the entire 12-month period of the intervention using stove use monitors (Digit-TL, LabJack Corporation). These devices recorded temperature readings every minute throughout the 12-month post-randomisation period. These data were used to determine which stoves were used in the household and the frequency and duration of cooking events^(12–14)^.

### Assessment of dietary intake

**Fig. 1:**
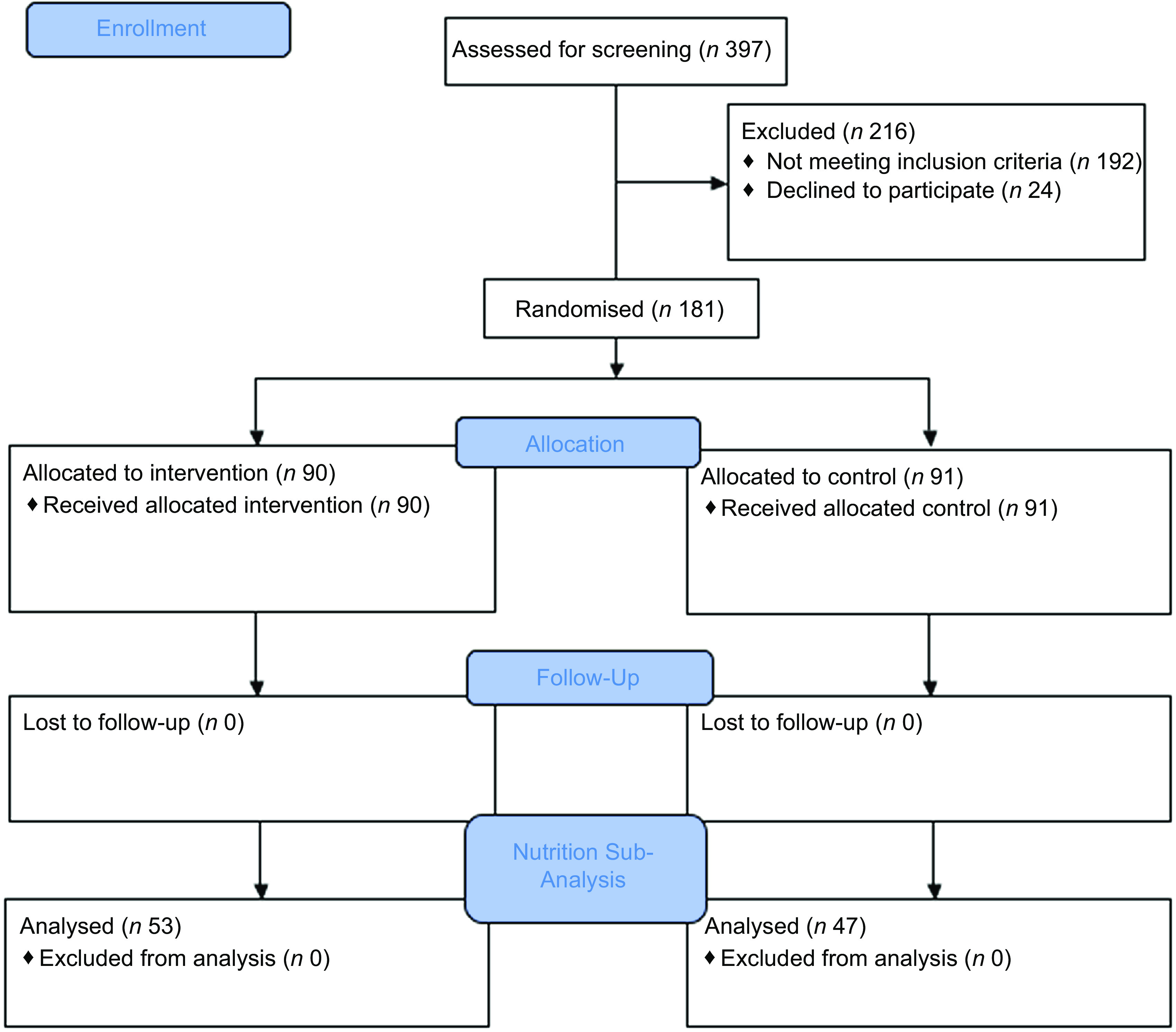
CONSORT flowchart: screening, randomisation and follow-up

We conducted a longitudinal assessment of dietary and Na intake in a randomly selected subset of 100 participants of the CHAP trial, including forty-seven participants in the control arm and fifty-three in the intervention arm (Fig. 1). None of the 100 participants in this subset were lost to follow-up during the 12-month study period. The measurements detailed below were conducted at baseline prior to randomisation, and at 6 months and 12 months after randomisation.

Dietary intake was assessed using 24-h dietary recalls administered at participants households by three local, licensed nutritionists. Dietary recalls were collected using the multiple-pass approach^(15,16)^, and field workers repeated the survey on three non-consecutive days, including 2 weekdays and 1 weekend day whenever possible. Field workers asked participants to list all foods and beverages they had eaten in the previous day. For multi-ingredient preparations, participants indicated the type and quantity of all ingredients used to prepare the dish and the size of the portion they had consumed. Field workers used direct weight when food items regularly used or leftovers from previous day were available, and visual methods to assess ingredient quantities using Henkel Max calibrated scales (BRD08-5KF Henkel AG & Co. KGaA) and a guide indicating Peruvian standardised portion sizes were previously developed by Asociación Benéfica PRISMA^(17)^. We did not assess the use of supplements.

In conjunction with every 24-h recall questionnaire, field workers administered a supplementary questionnaire that collected information on food allergies, whether or not food consumption during the recall period was representative of usual intake, and any illness during the recall period that could have affected the habitual dietary intake. Dietary 24-h recall questionnaires were collected using paper forms, given the large variety in types of meals and ingredients consumed.

Furthermore, Na intake was assessed using one 24-h urine sample from each participant at baseline, and at 6-months and 12-months post-randomisation. Na content in urine was chosen over dietary questionnaires to prevent under-reporting and other challenges on quantifying the salt in each recipe, added when cooked or processed food^(18)^. Briefly, field workers gave participants a bucket with a lid and instructed participants to collect all urine voided over a full 24 h in the bucket. Field workers returned the following day to collect the bucket. Urine samples were transported at 4°C from the field to the office, where field workers took an aliquot of 5 ml. Samples were shipped to a certified laboratory for processing (SynLab).

Body weight was measured in kilograms at baseline and 12 months post-randomisation using the SECA 220089 scale (SECA GMBH & Co) with a precision of ± 100 g, along with standing height and sitting height. At each time point, each measurement was taken 3 times and averaged to obtain a final value. Body weight scales were calibrated weekly. We used height and weight to calculate the BMI in kg/m^2^ for each participant.

At baseline, field workers also administered a questionnaire to collect demographic information including age, education level, main occupation, monthly income and asset ownership. Wealth quintiles were calculated from these variables, as an indicator of socioeconomic status, based on previous national surveys as previously described^(19)^.

Surveys were conducted on tablets using the Research Electronic Data Capture (REDCap) software (Vanderbilt University Medical Centre).

### Dietary and Na intake analysis

Dietary intake data from the three non-consecutive recalls were entered electronically into an in-house developed software database in Access (Microsoft Corp.), which calculated the participant’s individual daily intake of each food item for each day. Waste and raw-cooked conversion factors were applied to obtain a net weight of each food item registered in the recall surveys^(20)^. Nutritional composition of all foods consumed was calculated by linking the net grams of each ingredient with the corresponding nutritional values from the Peruvian Nutritional Composition Table^(21)^, and final nutrients intake per participant were averaged, which was the final one used as a common daily intake. Energy in kcal units were converted into kJ.

Food items were classified into ten food groups, based on those identified in the Minimum Dietary Diversity for Women guide,^(22)^ and we calculated a dietary diversity score for each participant, where the higher score indicates the more diverse diet. Furthermore, we calculated the quantity of food consumed within each food group to understand the sources of nutrients.

Urine samples were analysed using the ion-selective electrode method to determine the mEq of Na/l of urine in 24 h^(23)^. We lost six urine samples at baseline and one sample from 12 months post-randomisation due to logistical issues.

### Biostatistical methods

We calculated descriptive statistics of study groups at baseline and tested for differences between groups using two sample *t*-student and Mann–Whitney tests for continuous variables and chi-squared tests for categorical variables. Data distribution was analysed using histograms and the Shapiro–Wilk test for normality. With 100 participants, we estimated that we could detect a difference of 1046 kJ (250 kcal) of energy intake and 1 g of sodium intake between arms with 80 % power and 95 % confidence.

We adjusted macronutrients by total energy intake at baseline and follow-ups visits using the nutrients residual method, considering that energy intake was a potential confounder of the association between macronutrients and our intervention^(24)^. Participants’ data were defined as outliers if the mean energy of their three non-consecutive 24-h recalls at each follow-up visit was below 2510 kJ (600 kcal) or above 16 736 kJ/d (4000 kcal/d), based on outlier definitions from previous research^(25)^. We conducted sensitivity analyses with and without outliers to determine the effect of any outliers on our results. We also compared participants Na intake from 24-h urine results against the WHO guideline of 2 g/d^(26)^.

For our primary analysis, we compared 6- and 12-month post-randomisation average dietary intake of energy, macronutrients and Na between control and intervention arms, using two-sample *t*-test in intention-to-treat analyses. We also compared mean food group intake between study arms at 6- and 12-months post-randomisation using two-sample *t*-tests at the 5 % significance level.

Data analyses were performed using STATA 15 (Stata Corp.) and visualisations were made in R^(27)^.

## Results

### Participant characteristics

A total of 100 participants were enrolled in the ancillary study (Fig. 1). We summarise the characteristics of the forty-seven controls and fifty-three intervention participants in Table 1. At baseline, participants had an average age of 48·5 years (sd 10·2) and an average BMI of 26·8 kg/m^2^ (sd 4·3). Only one participant reported an underlying health condition (high cholesterol). None of the participants reported taking medications that could affect their dietary intake. Baseline dietary data was collected across three different seasons, pre-harvest (*n* 33), harvest (*n* 25) and post-harvest (*n* 42), with all three seasons represented equally across study groups (Table 1).

**Table 1:** Participant characteristics by trial arm at baseline

	Control (*n* 47)		Intervention (*n* 53)		Total (*n* 100)	
	Mean	sd	Mean	sd	Mean	sd
Age in years	47·4	11·9	49·5	8·5	48·5	10·2
BMI in kg/m^2^	26·7	4·1	26·9	4·5	26·8	4·3
	%	*n*	%	*n*	%	*n*
Wealth index quintiles						
Poorest	59·6	28	50·9	27	55	55
Poor	34·0	16	39·6	21	37	37
Middle	6·4	3	9·4	5	8	8
BMI categories						
Underweight	2·1	1	1·9	1	2·0	2
Normal	34·0	16	35·9	19	35·0	35
Overweight	44·7	21	39·6	21	42·0	42
Obesity	19·2	9	22·6	12	21·0	21
Harvest season						
Pre-harvest	29·8	14	35·9	19	33	33
Harvest	21·3	10	28·3	15	25	25
Post-harvest	48·9	23	35·9	19	42	42
Owned LPG stove						
No	23·5	12	28·3	15	27·0	27
Yes	74·5	35	71·7	38	73	73

### Dietary and Na intake at baseline

Mean daily energy intake was 8576·9 kJ (sd 2588·8) at baseline. Mean daily intake of protein was 55·6 g (sd 21·3), carbohydrates 372 g (sd 116·6) and fat 38·6 g (sd 18·5). Mean Na intake was 4·8 g (sd 1·9) (Table 2).

**Table 2:** Daily dietary intake by trial arm at baseline

	Control			Intervention			Total			Difference	*P*-value
	*n*	Mean	sd	*n*	Mean	sd	*n*	Mean	sd		
Energy (kJ/d)	47	8894·3	2762·2	53	8295·5	2416·2	100	8576·9	2588·8	598·8	0·25
Macronutrients adjusted by energy											
Protein (g/d)	47	57·0	13·6	53	54·6	15·1	100	55·6	14·4	2·4	0·19
Fat (g/d)	47	39·8	14·2	53	39·5	12·8	100	39·6	13·4	0·3	0·99
Carbohydrates (g/d)	47	370·8	129·7	53	373·3	34·3	100	372·1	34·0	−2·5	0·71
Na (g/d)	44	4·8	1·9	50	4·9	2·0	94	4·8	1·9	−0·1	0·89

At baseline, energy intake and Na intake were not significantly different between intervention and control participants (Table 2). Likewise, intake by food group and dietary diversity was similar between intervention and control participants at baseline (Table 3).

**Table 3:** Daily food group intake by trial arm at baseline

Food group (g/d)	Control			Intervention			Total			Difference	*P*-value
	*n*	Mean	sd	*n*	Mean	sd	*n*	Mean	sd		
Grains and roots	47	670·5	304·2	53	606·4	233·2	100	636·5	269·5	64·1	0·44
Pulses	47	36·2	39·7	53	33·7	34·1	100	34·9	36·6	2·5	0·98
Nuts and seeds	47	0		53	0·03	0·2	100	0·02	0·1	−0·03	0·18
Meat, poultry and fish	47	54·4	46·6	53	49·7	46·1	100	51·9	46·1	4·7	0·42
Eggs	31	20·9	20·9	30	21·7	15·0	61	21·3	18·1	−0·8	0·42
Dairy	31	89·9	133·8	30	42·1	65·1	61	64·5	105·5	47·1	0·12
Dark green leafy vegetables	31	12·1	17·2	30	5·6	6·6	61	8·6	13·1	6·5	0·05
Other vitamin A-rich fruits and vegetables	31	48·9	43·0	30	41·6	40·1	61	45·1	41·4	2·9	0·32
Other vegetables	31	40·4	26·8	30	37·5	29·6	61	38·9	28·2	7·4	0·33
Other fruits	31	63·9	57·4	30	66·9	57·7	61	65·5	57·3	−2·9	0·78
Minimum dietary diversity in women (MDD-W)											
Minimum Dietary Diversity in Women score	31	7·72	1·3	30	7·38	1·0	61	7·54	1·2	0·3	0·15
	*n*	%	*n*	*n*	%	*n*	*n*	%	*n*		
Minimum dietary diversity in women (> 5 groups)	31	97·8	46	30	100	53	61	99·0	99	–	0·29

### Post-intervention intake

**Fig. 2:**
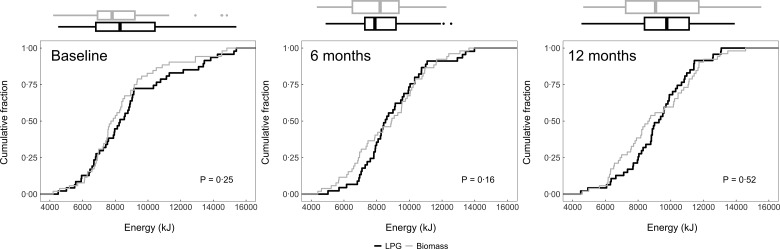
Empirical cumulative distribution functions and boxplots of daily energy intake by visit and trial arm: LPG (intervention) and biomass (control)

**Fig. 3:**
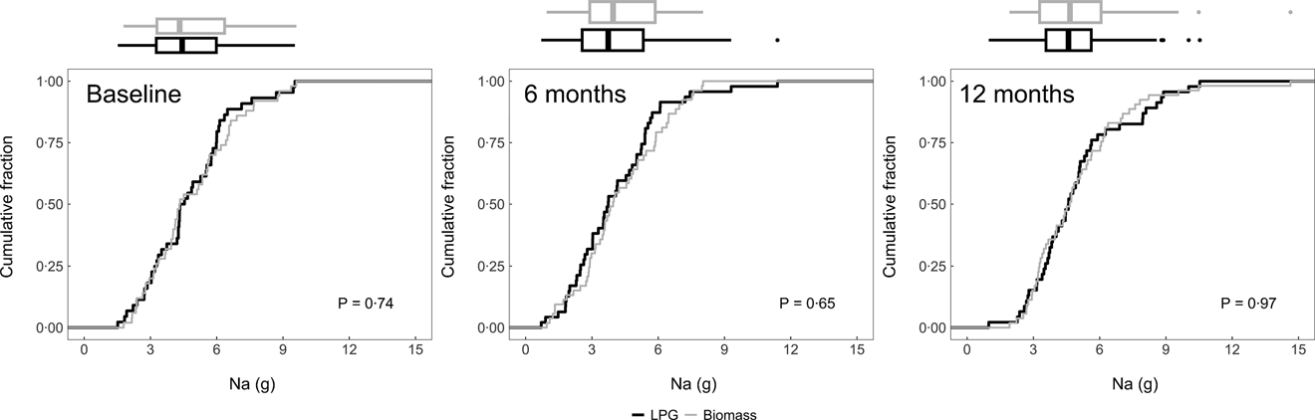
Empirical cumulative distribution functions and boxplots of daily Na intake by visit and trial arm: LPG (intervention) and biomass (control)

**Fig. 4:**
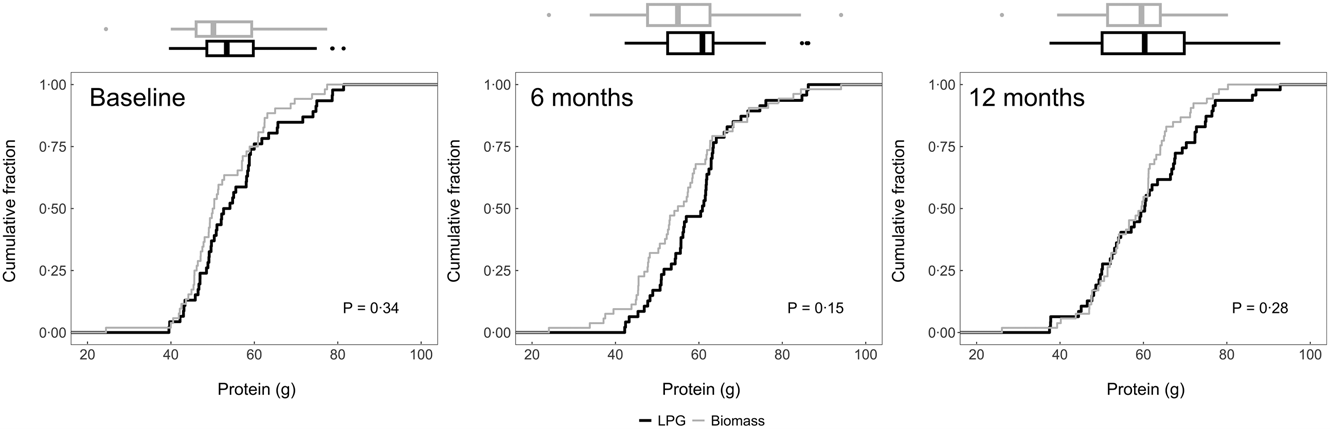
Empirical cumulative distribution functions and boxplots of daily protein consumption adjusted for energy intake by visit and trial arm: LPG (intervention) and biomass (control)

**Fig. 5:**
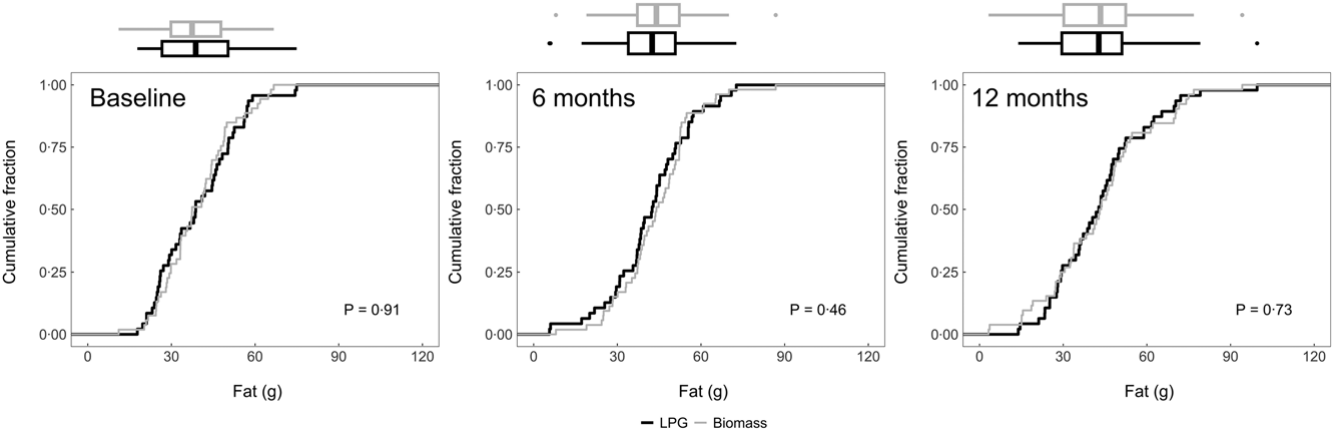
Empirical cumulative distribution functions and boxplots of daily fat consumption adjusted for energy intake by visit and trial arm: LPG (intervention) and biomass (control)

**Fig. 6:**
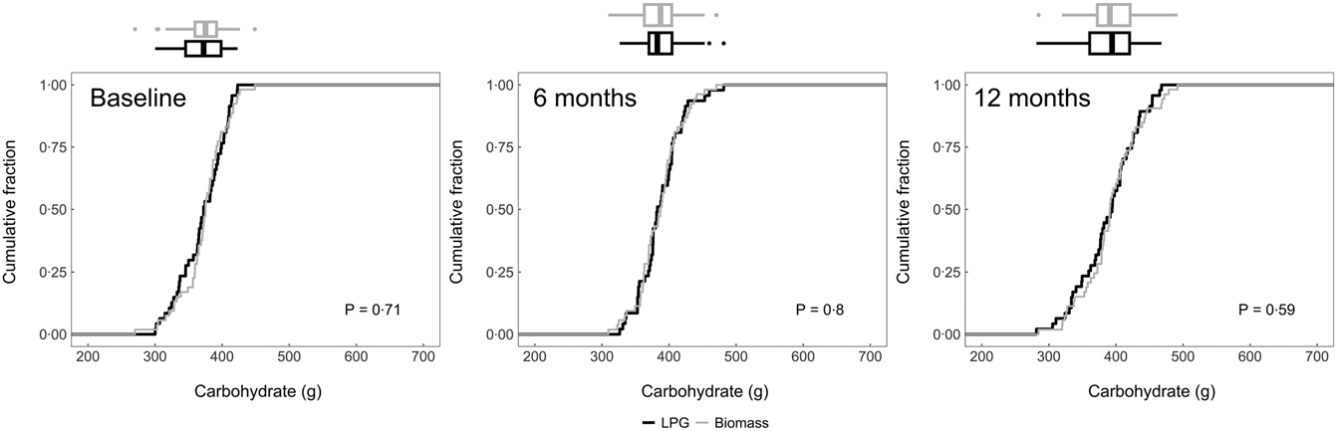
Empirical cumulative distribution functions and boxplots of daily carbohydrate intake adjusted for energy intake by visit and trial arm: LPG (intervention) and biomass (control)

We plotted the cumulative distribution functions of daily dietary energy (Fig. 2), Na (Fig. 3), protein (Fig. 4), fat (Fig. 5) and carbohydrates (Fig. 6) intakes at baseline, 6-month and 12-month visits for each study arm. There were no clear differences in dietary or Na intake between study groups at baseline or any time post-randomisation. Indeed, average daily energy intake (averaged across the 6- and 12-month follow-up visits) was not significantly different between control and intervention arms (9292·4 kJ *v.* 8788·3 kJ, *P* = 0·22) (Table 4). Average macronutrient intakes were also not significantly different between control and intervention arms: protein (60·2 g *v*. 57·2 g, *P* = 0·09), fat (42·7 g *v*. 43·2 g, *P* = 0·86) and carbohydrates (388·1 g *v*. 389·6 g, *P* = 0·79). Finally, average Na urine levels (averaged across the 6- and 12-month follow-up visits) did not differ between control and intervention arms (4·5 g *v*. 4·6 g, *P* = 0·79).

**Table 4:** Average daily energy, macronutrient and Na intake by trial arm, averaging 6- and 12-month post-randomisation measurements by participant

	Control			Intervention			Total			Difference	*P*-value
	*n*	Mean	sd	*n*	Mean	sd	*n*	Mean	sd		
Energy (kJ/d)	47	9292·4	2045·3	53	8788·3	2066·8	100	9025·2	2061·9	504·2	0·22
Macronutrients adjusted by energy											
Protein (g/d)	47	60·2	9·3	53	57·2	8·9	100	58·6	9·2	3	0·09
Fat (g/d)	47	42·7	13·0	53	43·2	11·6	100	42·9	12·2	−0·5	0·86
Carbohydrates (g/d)	47	388·1	30·7	53	389·6	27·7	100	388·9	28·9	−0·8	0·79
Na (g/d)	46	4·5	1·7	53	4·6	1·5	99	4·6	1·6	−0·1	0·79

### Intake by food group

During the post-randomisation period, there were no statistically significant differences between control and intervention arms for most food groups: grains and roots (645·9 g *v*. 617·6 g, *P* = 0·47), pulses (33·9 g *v*. 25·8 g, *P* = 0·12), nuts and seeds (0·002 g *v*. 0·009 g, *P* = 0·51), meat, poultry and fish (58·7 g *v*. 55·4 g, *P* = 0·63), eggs (31·3 g *v*. 24·8 g, *P* = 0·39), dark green leafy vegetables (8·3 g *v*. 9·7 g, *P* = 0·62), other vitamin A-rich fruits and vegetables (49·8 g *v*. 54·1 g, *P* = 0·47) and other vegetables (41·7 g *v*. 35·4 g, *P* = 0·20). However, control participants consumed more dairy (104·9 g *v*. 61·6 g, *P* = 0·04) and fruits (61·4 g *v*. 43·8 g, *P* = 0·05) than intervention participants. Dietary diversity scores averaged across the 6- and 12-month follow-up visits (7·4 *v*. 7·1, *P* = 0·22) were also not significantly different between control and intervention participants.

### Sensitivity analyses

At baseline, there were no significant differences in age (*P* = 0·52), BMI (*P* = 0·99), wealth quintile (*P* = 0·28) and LPG ownership (*P* = 0·91) between participants who were part of the nutrition subgroup (*n* 100) and those who were part of the parent CHAP trial but were not included in this study subset (*n* 80).

We identified two outlier observations with energy intakes above 16 736 kJ (4000 kcal) at the 6-month follow-up and performed the primary analysis with and without these outliers. Energy intake was not statistically different between control and intervention arms in analysis when outliers were included (9292·4 kJ *v.* 8788·3 kJ, *P* = 0·22) or excluded (9011·9 kJ *v.* 8788·3 kJ, *P* = 0·55). Likewise, Na urine levels was not different between control and intervention arms with outliers (4·5 g *v*. 4·6 g, *P* = 0·79) or without outliers (4·6 g *v*. 4·6 g, *P* = 0·87).

Additionally, we conducted an analysis in which we included only those participants in the control arm who used their biomass-burning stoves for more than 50 % of their cooking time and participants in the intervention arm who used LPG stoves for more than 95 % of their cooking minutes. Differences in average energy intake between control and intervention participants (averaged across the 6- and 12-month visits) remained non-significant (9984·4 kJ *v.* 8962·4 kJ, respectively; *P* = 0·16) as did Na intake (4·3 g *v*. 4·8 g, respectively; *P* = 0·35).

## Discussion

In this randomised controlled trial, we found that adult women in rural Puno, Peru, assigned to use an LPG stove, continuous fuel distribution and behavioural messaging did not have differences in daily energy, macronutrient or Na intake over 1 year when compared to controls.

Contrary to our findings, Anderman *et al.* found that participants who cooked with biogas stoves had more dietary diversity than those cooking with biomass stoves, while we found that dairy and fruit intake was lower in the LPG intervention group^(6)^, this difference can be due to the lack of cooking for these food groups. However, the Dietary Diversity Score for Women^(22)^ applied by Anderman *et al.* only considered the presence or absence of the ten food groups, while our study compared the actual quantities consumed as well. Detecting significant differences between small quantities of foods consumed may be less likely to show significant differences than assessing for the presence or absence of the food but may allow for a more meaningful comparison. A lack of difference in dietary consumption between intervention and control groups could be related to the fact that participants in both groups had a low socio-economic status. Other research has suggested that dietary intake is significantly affected by socio-economic status^(28,29)^. This suggests that changes in dietary intake may be driven more by wealth status than the type of stove used.

This study showed that the transition from biomass to LPG stoves did not affect the dietary and Na intake in participants with similar socio-economic status. For example, we did not find that adoption of LPG resulted in increased Na consumption, which suggests that participants did not add salt to food cooked with LPG, for example, to make it taste better. We also did not find that transitioning to LPG increased total energy consumption, suggesting that participants did not prepare more food, given, for example, the increased ease or speed of food preparation. All intervention participants received a cooking demonstration before their LPG stove was installed, which may have helped them learn how to prepare traditional dishes in the same way that they did with their biomass stove. These findings suggest that the adoption of LPG by poor, rural, previous biomass users did not negatively or positively affect dietary habits.

Furthermore, our study contributes information about the dietary and Na intake of women in high-altitude and rural settings of Peru which is not well-characterised in the literature. The prevalence of anaemia in children under 5 years of age in Puno is the highest in Peru^(30)^. Understanding the dietary habits of adult women, who are often responsible for cooking and feeding their families, can inform the design of interventions to improve family diets and subsequently decrease anaemia among children.

Our study has some strengths. The randomised nature of our trial ensured that intervention and control participants had similar characteristics, thus allowing us to compare diet between primary LPG users and primary biomass users without potential biases from confounders. By providing free LPG fuel for 1 year, we were able to assess dietary impacts of the LPG stove over time without the common challenge that LPG users are typically wealthier than non-users. We also observed high compliance with LPG stove use among our intervention participants^(14)^, thus allowing us to see how near-complete transition from one stove to another affected diet. Our study additionally accounted for seasonality by conducting measurements across seasons, thus reducing the potential impact of differential food availability by season on dietary consumption. In addition to this, our study used biological samples to assess Na intake, thus avoiding challenges with self-reported Na consumption^(18)^.

There are also some potential shortcomings. First, dietary intake was self-reported in 24-h recall surveys. Our participants, who had a high average BMI, may have underreported their dietary intake, a problem that is commonly seen in participants with high BMI^(31)^. Second, self-reported data may also be affected by recall bias, in which participants forget what they ate or drank in the previous day, or social desirability bias, in which participants report eating foods perceived to be healthier to please the fieldworkers. These biases may have influenced the accuracy of estimated energy and micronutrient intakes^(31)^. Despite these potential limitations, 24-h recalls are one of the most common survey methods due to the detailed information provided for different purposes. Third, nutrient estimation was not adjusted by the retention factor according to the cooking method^(32)^, which may have allowed us to obtain more precise estimates. There may be a concern that our estimated differences in dietary and Na intake may have been large. However, a 1046 kJ (250 kcal) difference represents around the 11 % of the daily energy intake in our sample size at baseline, and, overall, it is less than 10 % of the general recommendation of energy intake in adults^(33)^. Second, a 1 g difference of Na intake represents about 20 % of the intake in our population at baseline, and it is less than a 3·2 g difference had we used the ideal limit of 1·5 g recommended by the American Heart Association (AHA) for heart health^(34)^. Following AHA guidelines, even cutting back salt intake by 1 g per day can improve blood pressure^(34)^, which was one of the primary outcomes of the trial^(13)^. However, smaller effects for both energy and Na intake may still be relevant and may go undetected.

Our research focussed on dietary intake among participants who were not paying for cooking fuel (LPG users received free fuel from the trial, and biomass users collected free biomass fuel). Further research is needed on dietary patterns when people must pay for their own LPG to understand how LPG purchases may affect the household’s ability to purchase food items and subsequently their dietary intake. Future studies should consider the potential impact of nutritional differences between cleaner fuels and biomass users when evaluating the impact of cleaner fuel interventions on cardiovascular and pulmonary health outcomes.

Our intervention with LPG stoves did not affect dietary and Na intake within a cleaner cooking intervention trial. Our findings suggest that the promotion of LPG stoves should not be limited by concerns over potential negative dietary impacts, when implemented in a manner similar to this study. However, nutritional improvements are also unlikely to occur. Nutritional education paired with LPG stove introduction may be necessary to achieve both HAP reductions and increased adherence to nutritional guidelines to achieve maximum health benefits.
